# First genomic analysis of a strain of *Ralstonia pseudosolanacearum* isolated from Mayotte island

**DOI:** 10.1186/s12863-025-01395-2

**Published:** 2025-12-08

**Authors:** Eva Caly-Simbou, Marie Veronique Nomenjanahary, Stéphanie Javegny, Claudine Boyer, Jean-Jacques Chéron, Stéphane Ramin-Mangata, Stéphane Poussier, Yann Pecrix

**Affiliations:** 1 University of Reunion Island, UMR PVBMT, Saint-Pierre, Reunion Island 97410 France; 2 CIRAD, UMR PVBMT, Saint-Pierre, La Réunion, F-97455 France

**Keywords:** *Ralstonia solanacearum* species complex, South-West Indian Ocean, Whole-genome sequencing, Complete genome sequence

## Abstract

**Objectives:**

The *Ralstonia solanacearum* species complex (*RSSC*) encompasses phytopathogenic bacteria responsible for bacterial wilt, a devastating disease affecting a wide range of agriculturally important crops. In the South-West Indian Ocean, lineage I-18 of *R. pseudosolanacearum* has emerged as a particularly destructive pathogen, posing a serious threat to regional food security. In this context, we report the complete genome sequence of isolate RUN2161, collected in Mayotte. This first genome from this island provides a valuable resource for unraveling the evolutionary and epidemiological mechanisms driving the emergence and spread of highly epidemic strains in agriculture..

**Data description:**

The genome of strain RUN2161 from Mayotte was sequenced using Illumina short reads and Nanopore long reads. A hybrid assembly was performed resulting in a complete genome of 5,989,529 bp with a G + C content of 66.7%. Functional annotation identified 5,268 CDS, 12 rRNAs, 61 tRNA genes, and 4 ncRNAs, assembled into one chromosome, one megaplasmid and one plasmid. Accessory plasmids are uncommon in *RSSC*. The RUN2161 plasmid contains Type IV secretion system genes, commonly found on conjugative plasmids, but less commonly, it also carries Type II secretion system genes involved in secretion of toxins and degradative enzymes, which could contribute to epidemiological success.

## Objective

The *Ralstonia solanacearum* species complex (*RSSC*), responsible for bacterial wilt in many economically significant crops is distributed globally [1]. *RSSC* is a species complex divided into species and phylotypes, corresponding to their geographical origins: *R. pseudosolanacearum* from Asia (Phylotype I) and Africa (Phylotype III), *R. solanacearum* from Americas (Phylotype IIA and IIB), and *R. syzygii* from Indonesia-Australia (Phylotype IV) [2–4]. Within these phylotypes, 71 sequevars, based on the similarities in their nucleic sequence that encode egl gene have been defined so far [5].

In the South-West Indian Ocean region, the epidemiological distribution of the *RSSC* is particularly noteworthy. While phylotype I is dominant in this area [6], distinct prevalence patterns have been reported between sequevars I-18 and I-31. While I-31 strains are predominant in the small islands of this region as well as in East Africa [6–8], I-18 strains are strongly established in Madagascar [9, 10]. Interestingly, recent study highlights genomic diversity within the sequevar I-18 [10, 11].

In Mayotte Island, sequevar I-18 strains have been detected in agricultural fields, raising new concerns for local crop production [7]. Here, we present the first chromosome-level hybrid assembly of a Mahoran I-18 strain, isolated in 2012 from *Solanum lycopersicum* (tomato), in Dembeni in the east coast of Mayotte.

## Data description

High molecular weight genomic DNA of *R. pseudosolanacearum* RUN2161 strain, was extracted using a protocol adapted for *RSSC* [11]. Samples were sequenced by the Genewiz-Azenta Laboratory platform (Leipzig, Germany) using Illumina NovaSeq technology. Long-read sequencing was performed using an R10.4.1 MinION flowcell and the SQK-RBK 114.96 Rapid Barcoding Kit (Oxford Nanopore Technology, UK). Basecalling was conducted with Dorado v0.9.0 [12] in the super accuracy mode. The genome assembly was performed using a hybrid pipeline [13]. Short reads and long reads were trimmed using Trimmomatic v0.39 [14] and Filtlong v0.2.1 [12], respectively. An initial hybrid assembly was generated with Unicycler v0.5.1 [15]. The assembly was first polished with long reads using four iterations of Racon v1.4.3 [16], followed by a second long-read polishing with Medaka v1.9.1 [17]. A third polishing step using short reads was performed with four iterations of NextPolish [18]. Finally, circularization of the replicons was performed using Berokka v0.2.3 [12].

The complete genome assembly (DataSet 3; GCA_052747835.1), has a size of 5,989,529 bp. Assembly metrics and quality control, performed using Quast v.5.2.0 [19], BUSCO v.5.8.3 [20] and CheckM v.1.2.2 [21], revealed the high quality of this assembly with a very high level of completeness (99.8%) and no evidence of gene duplication or contamination (DataFile 1). The functional annotation was performed using the NCBI PGAP pipeline [22]. The entire genome encodes 5,161 coding genes and 77 RNAs (including 12 rRNAs, 61 tRNAs, and 4 ncRNAs) and comprise a circular chromosome of 3,758,906 bp with 66.7% GC content and 3,403 coding genes, a megaplasmid of 2,178,702 bp with 66.5% GC content and 1,692 coding genes, and a plasmid of 51,921 bp with 61.5% GC content and 66 coding genes (DataSet 3, DataFile 2).

While a megaplasmid is consistently found across RSSC, accessory plasmids are not ubiquitous and majority of those described are small (< 50 kb) [23, 24], although larger plasmids over 100 kb can also be found [25]. We screened the RUN2161 plasmid sequence against 864 *RSSC* genomes available in the NCBI database using BLASTN. Ten significant matches were identified, showing 63–87% nucleotide identity. Nine of these plasmids originated from phylotype I strains from China, South Korea, Thailand, and Israel, while one match corresponded to a phylotype IV strain from Indonesia (DataFile 3). The RUN2161 plasmid contains 66 genes, of which 41% correspond to hypothetical proteins with no assigned function. We reported Type IV secretion system (T4SS) genes and a toxin-antitoxin system that are commonly found on conjugative plasmids (DataFile 2).

Surprisingly, the plasmid also carries three proteins associated with the type II secretion system (T2SS) of which an ATPase and a GspD protein. T2SS have been described as a major element implicated in toxins delivery including hydrolytic enzymes and toxins, including proteases, lipases. It often includes a cytoplasmic ATPase [26]. Interestingly, we have found two lytic transglycosylases, a phospholipase D and a peptidase S24. These enzymes, potentially secreted by the T2SS, are thus high valuable candidates to study the molecular mechanisms of virulence and epidemic success.

**Table 1:** Overview of data files/data sets

Label	Name of data file/data set	File types (file extension)	Data repository and identifier (DOI or accession number)
DataSet 1	Raw short Illumina sequencing reads 1 and 2	Sequence file (.fastq)	NCBI Sequence Read Archive (https://identifiers.org/ncbi/insdc.sra:SRR35491076) [27]
DataSet 2	Raw long nanopore sequencing reads	Sequence file (.fastq)	NCBI Sequence Read Archive (https://identifiers.org/ncbi/insdc.sra:SRR35404195) [28]
DataSet 3	Complete genome and annotation of *R. pseudosolanacearum* RUN2161	GenBank/Annotation files	NCBI GenBank (http://identifiers.org/assembly:GCA_052747835.1) [29]
DataFile 1	Quality control of assembly (QUAST, BUSCO and CheckM)	MS Excel file (.xlsx)	Figshare, 10.6084/m9.figshare.30272398 [30]
DataFile 2	Circular visualization of RUN2161 genome	Portable Document Format file	Figshare, 10.6084/m9.figshare.30273385 [31]
DataFile 3	Alignments of plasmids	Portable Document Format file	Figshare, 10.6084/m9.figshare.30273451 [32]

## Limitations

This study provides a high-quality genome assembly, combining short reads, which offer low error rates, with long reads, which enhance contiguity. This approach provides the best genome quality achievable with available technologies. However, the high genetic diversity of strains circulating in Mayotte highlights the need to investigate other genomes to gain a more comprehensive understanding of variability within *RSSC*. Moreover, genome annotation still requires improvement, as 18% of the predicted proteins remain of unknown function. Finally, functional validation of these candidate genes will be essential to fully understand their biological roles and potential implications.

## Data Availability

The genomic data described in this Data note can be freely and openly accessed on GenBank of NCBI under Bioproject accession PRJNA1328457 and BioSample accessions SAMN51336171. Raw Illumina and Nanopore reads were deposited under NCBI SRA accessions SRR35491076 and SRR35404195. The results of genome analysis have been uploaded to the Figshare repository. Please see Table 1 for details and links to data.

## Abbreviations

RSSC: Ralstonia solanacearum species complex
T2SS: Type II secretion system
T4SS: Type IV secretion system

## References

[1] Lowe-Power TM, Avalos J, Bai Y, Munoz MC, Chipman K, Elmgreen VN, et al. A Meta-analysis of the known global distribution and host range of the ralstonia species complex. 2020. 10.1101/2020.07.13.189936

[2] Fegan M, Prior P. How complex is the ralstonia solanacearum species complex. Bacterial wilt disease and the ralstonia solanacearum species complex. APS; 2005.

[3] Prior P, Ailloud F, Dalsing BL, Remenant B, Sanchez B, Allen C. Genomic and proteomic evidence supporting the division of the plant pathogen ralstonia solanacearum into three species. BMC Genomics. 2016;17:90. 10.1186/s12864-016-2413-z. 26830494 10.1186/s12864-016-2413-z PMC4736150

[4] Safni I, Cleenwerck I, De Vos P, Fegan M, Sly L, Kappler U. Polyphasic taxonomic revision of the ralstonia solanacearum species complex: proposal to emend the descriptions of ralstonia solanacearum And ralstonia Syzygii And reclassify current R. Syzygii strains as ralstonia Syzygii subsp. Syzygii subsp. nov., R. solanacearum phylotype IV strains as ralstonia Syzygii subsp. Indonesiensis subsp. nov., banana blood disease bacterium strains as ralstonia Syzygii subsp. Celebesensis subsp. nov. And R. solanacearum phylotype I And III strains as ralstonia pseudosolanacearum sp. nov. Int J Syst Evol Microbiol. 2014;64:3087–103. 10.1099/ijs.0.066712-0. 24944341 10.1099/ijs.0.066712-0

[5] Cellier G, Gauche MM, Cheron JJ, Pecrix Y. How to conduct phylogenetic endoglucanase (egl) inference using the reference ralstonia solanacearum species complex curated database. Phytopathology. 2025;115:548–54. 10.1094/PHYTO-10-24-0318-SC. 39932454 10.1094/PHYTO-10-24-0318-SC

[6] Yahiaoui N, Chéron J-J, Ravelomanantsoa S, Hamza AA, Petrousse B, Jeetah R, et al. Genetic diversity of the ralstonia solanacearum species complex in the Southwest Indian ocean Islands. Front Plant Sci. 2017;8:2139. 10.3389/fpls.2017.02139. 29312394 10.3389/fpls.2017.02139 PMC5742265

[7] Chesneau T, Maignien G, Boyer C, Chéron J-J, Roux-Cuvelier M, Vanhuffel L, et al. Sequevar diversity and virulence of ralstonia solanacearum phylotype I on Mayotte Island (Indian Ocean). Front Plant Sci. 2018;8:2209. 10.3389/fpls.2017.02209. 29354148 10.3389/fpls.2017.02209 PMC5760537

[8] Cellier G, Nordey T, Cortada L, Gauche M, Rasoamanana H, Yahiaoui N, et al. Molecular epidemiology of *Ralstonia pseudosolanacearum* phylotype I strains in the Southwest Indian ocean region and their relatedness to African strains. Phytopathology^®^. 2023;113:423–35. 10.1094/PHYTO-09-22-0355-R. 36399027 10.1094/PHYTO-09-22-0355-R

[9] Rasoamanana H, Ravelomanantsoa S, Yahiaoui N, Dianzinga N, Rébert E, Gauche M-M, et al. Contrasting genetic diversity and structure among Malagasy ralstonia pseudosolanacearum phylotype I populations inferred from an optimized multilocus variable number of tandem repeat analysis scheme. PLoS ONE. 2020;15:e0242846. 10.1371/journal.pone.0242846. 33290390 10.1371/journal.pone.0242846 PMC7723262

[10] Rasoamanana H, Ravelomanantsoa S, Nomenjanahary M-V, Gauche M-M, Prior P, Guérin F, et al. Bacteriocin production correlates with epidemiological prevalence of phylotype I Sequevar 18 ralstonia pseudosolanacearum in Madagascar. Appl Environ Microbiol. 2023;0:e01632–22. 10.1128/aem.01632-22. 10.1128/aem.01632-22 PMC9888187 36602304

[11] Nomenjanahary MV, Rasoamanana H, Javegny S, Rieux A, Ravelomanantsoa S, Poussier S, et al. High-Quality genome resource of eight strains of ralstonia pseudosolanacearum causing bacterial wilt in Madagascar. PhytoFrontiers^™^. 2025;5:94–9. 10.1094/PHYTOFR-10-24-0110-A.

[12] Oxford Nanopore Technologies. Dorado. GitHub repository GitHub Available online at: https://github.com/nanoporetech/dorado Accessed 10 Sept 2025.

[13] Luan T, Commichaux S, Hoffmann M, Jayeola V, Jang JH, Pop M, et al. Benchmarking short and long read Polishing tools for nanopore assemblies: achieving near-perfect genomes for outbreak isolates. BMC Genomics. 2024;25:679. 10.1186/s12864-024-10582-x. 38978005 10.1186/s12864-024-10582-x PMC11232133

[14] Bolger AM, Lohse M, Usadel B. Trimmomatic: a flexible trimmer for illumina sequence data. Bioinformatics. 2014;30:2114–20. 10.1093/bioinformatics/btu170. 24695404 10.1093/bioinformatics/btu170 PMC4103590

[15] Wick RR, Judd LM, Gorrie CL, Holt KE, Unicycler. Resolving bacterial genome assemblies from short and long sequencing reads. PLoS Comput Biol. 2017;13:e1005595. 10.1371/journal.pcbi.1005595. 28594827 10.1371/journal.pcbi.1005595 PMC5481147

[16] Vaser R, Sović I, Nagarajan N, Šikić M. Fast and accurate de Novo genome assembly from long uncorrected reads. Genome Res. 2017;27:737–46. 10.1101/gr.214270.116. 28100585 10.1101/gr.214270.116 PMC5411768

[17] Wright C, Wykes M. Medaka: sequence correction provided by ONT research. Medaka: sequence correction provided by ONT research; 2023.

[18] Hu J, Fan J, Sun Z, Liu S. NextPolish: a fast and efficient genome Polishing tool for long-read assembly. Bioinformatics. 2020;36:2253–5. 10.1093/bioinformatics/btz891. 31778144 10.1093/bioinformatics/btz891

[19] Gurevich A, Saveliev V, Vyahhi N, Tesler G. QUAST: quality assessment tool for genome assemblies. Bioinformatics. 2013;29:1072–5. 10.1093/bioinformatics/btt086. 23422339 10.1093/bioinformatics/btt086 PMC3624806

[20] Manni M, Berkeley MR, Seppey M, Simão FA, Zdobnov EM. BUSCO update: novel and streamlined workflows along with broader and deeper phylogenetic coverage for scoring of Eukaryotic, Prokaryotic, and viral genomes. Mol Biol Evol. 2021;38:4647–54. 10.1093/molbev/msab199. 34320186 10.1093/molbev/msab199 PMC8476166

[21] Parks DH, Imelfort M, Skennerton CT, Hugenholtz P, Tyson GW. CheckM: assessing the quality of microbial genomes recovered from isolates, single cells, and metagenomes. Genome Res. 2015;25:1043–55. 10.1101/gr.186072.114. 25977477 10.1101/gr.186072.114 PMC4484387

[22] Tatusova T, DiCuccio M, Badretdin A, Chetvernin V, Nawrocki EP, Zaslavsky L, et al. NCBI prokaryotic genome annotation pipeline. Nucleic Acids Res. 2016;44:6614–24. 10.1093/nar/gkw569. 27342282 10.1093/nar/gkw569 PMC5001611

[23] Aoun N, Georgoulis SJ, Avalos JK, Grulla KJ, Miqueo K, Tom C et al. A pangenomic atlas reveals eco-evolutionary dynamics that shape type VI secretion systems in plant-pathogenic ralstonia. mBio 15:e00323–24. 10.1128/mbio.00323-24 10.1128/mbio.00323-24 PMC11481896 39191402

[24] Remenant B, Coupat-Goutaland B, Guidot A, Cellier G, Wicker E, Allen C, et al. Genomes of three tomato pathogens within the ralstonia solanacearum species complex reveal significant evolutionary divergence. BMC Genomics. 2010;11:379. 10.1186/1471-2164-11-379. 20550686 10.1186/1471-2164-11-379 PMC2900269

[25] Ding S, Yu L, Lan G, Tang Y, Li Z, He Z, et al. Identification and genomic characterization of *Ralstonia pseudosolanacearum* strains isolated from Pepino melon in China. Physiol Mol Plant Pathol. 2023;125:101977. 10.1016/j.pmpp.2023.101977.

[26] Korotkov KV, Sandkvist M, Architecture, Function, and Substrates of the Type II Secretion System. EcoSal Plus 8:10.1128/ecosalplus.ESP-0034–2018. 10.1128/ecosalplus.esp-0034-2018 10.1128/ecosalplus.esp-0034-2018 PMC6638579 30767847

[27] Caly-Simbou et al. DataSet 1. Raw short Illumina sequencing reads 1 and 2 of *Ralstonia pseudosolanacearum* RUN2161. Available from: https://identifiers.org/ncbi/insdc.sra:SRR35491076

[28] Caly-Simbou et al. DataSet 2. Raw long nanopore sequencing reads of *Ralstonia pseudosolanacearum* RUN2161. Available from: https://identifiers.org/ncbi/insdc.sra:SRR35404195

[29] Caly-Simbou et al. DataSet 3. Complete genome and annotation of *Ralstonia pseudosolanacearum* RUN2161. Available from: http://identifiers.org/assembly:GCA_052747835.1

[30] Caly-Simbou et al. DataFile 1. Quality control of genome assembly of *Ralstonia pseudosolanacearum* RUN2161. Available from: 10.6084/m9.figshare.30272398

[31] Caly-Simbou et al. DataFile 2. Circular visualization of *Ralstonia pseudosolanacearum* RUN2161 genome. Available from: 10.6084/m9.figshare.30273385

[32] Caly-Simbou et al. DataFile 3. Alignments of plasmids. Available from: 10.6084/m9.figshare.30273385

